# Trends in Flexible Sensing Technology in Smart Wearable Mechanisms–Materials–Applications

**DOI:** 10.3390/nano15040298

**Published:** 2025-02-15

**Authors:** Sen Wang, Haorui Zhai, Qiang Zhang, Xueling Hu, Yujiao Li, Xin Xiong, Ruhong Ma, Jianlei Wang, Ying Chang, Lixin Wu

**Affiliations:** 1 School of Mechanical Engineering, Yancheng Institute of Technology, Yancheng 224051, China; 13052968098@163.com; 2 School of Automotive Engineering, Yancheng Institute of Technology, Yancheng 224051, China; 15651318378@163.com (Q.Z.); yujiaol20@mails.jlu.edu.cn (Y.L.); xiongxin@ycit.edu.cn (X.X.); 3 School of Materials Science and Engineering, Dalian University of Technology, Dalian 116024, China; yingc@dlut.edu.cn; 4 Fujian Institute of Research on the Structure of Matter, Chinese Academy of Sciences, Fuzhou 350002, China; huxueling@fjirsm.ac.cn (X.H.); lxwu@fjirsm.ac.cn (L.W.); 5 CAS Haixi Industrial Technology Innovation Center in Beilun, Ningbo 315830, China

**Keywords:** flexible sensors, sensing mechanisms, intelligent wearable devices, materials, health medical monitoring, human–computer interaction

## Abstract

Flexible sensors are revolutionizing our lives as a key component of intelligent wearables. Their pliability, stretchability, and diverse designs enable foldable and portable devices while enhancing comfort and convenience. Advances in materials science have provided numerous options for creating flexible sensors. The core of their application in areas like electronic skin, health medical monitoring, motion monitoring, and human–computer interaction is selecting materials that optimize sensor performance in weight, elasticity, comfort, and flexibility. This article focuses on flexible sensors, analyzing their “sensing mechanisms–materials–applications” framework. It explores their development trajectory, material characteristics, and contributions in various domains such as electronic skin, health medical monitoring, and human–computer interaction. The article concludes by summarizing current research achievements and discussing future challenges and opportunities. Flexible sensors are expected to continue expanding into new fields, driving the evolution of smart wearables and contributing to the intelligent development of society.

## 1. Introduction

In recent years, the field of flexible electronics has been experiencing rapid growth, with intelligent wearable electronic devices garnering significant attention from researchers worldwide in areas such as electronic skin, health monitoring, motion tracking, smart healthcare, and human–computer interaction [1,2,3,4,5]. For these applications, intelligent wearable sensors should exhibit fundamental characteristics such as high sensitivity, excellent flexibility, optimal stretchability, and robust stability [6,7,8,9,10]. The array of flexible sensors includes, but is not limited to, flexible resistive sensors [11], flexible piezoelectric sensors [12], flexible capacitive sensors [13], and flexible triboelectric sensors [14], each operating on distinct sensing mechanisms. However, their core principle is to convert physical and chemical signals into electrical signals, process those signals, and then output them into wearable devices for monitoring [15,16]. Physical quantity sensors are designed to monitor various physical signals of the human body, such as heart rate, blood pressure, pulse, and breathing [17]. These sensors play a crucial role in capturing and converting mechanical deformation into electrical signals, a process that is characterized by its simplicity and high sensitivity. The applications of this sensing form will be elaborated in Chapter 5 of this paper. Chemical quantity sensors are primarily employed to detect biochemical substances in the human body, such as glucose and lactic acid present in sweat, saliva, and interstitial fluid [18]. These sensors typically operate on electrochemical principles, leveraging the chemical reactions between the electrode and the target analytes to generate measurable electrical signals. Biosensors integrate biometric elements, such as enzymes, nucleic acids, and antibodies, that enable specific recognition of target analytes. By combining these biometric elements with physicochemical transducers, biosensors are capable of converting biological recognition events into measurable signals with high selectivity and sensitivity [19]. Early wearable sensors primarily targeted motion monitoring, such as step counting and heart rate tracking. In recent years, driven by advancements in materials science, electronics, and microfabrication technologies, wearable sensors have evolved towards miniaturization, flexibility, low power consumption, and intelligence. Meanwhile, the limitations of single-signal monitoring have become increasingly evident, and multi-channel wearable sensing systems are emerging as the future development trend. The current research emphasis in the realm of flexible sensors is on the relentless exploration and development of innovative flexible materials. Furthermore, through material composite technology, the integration of various materials’ strengths can be achieved, leading to the fabrication of flexible sensors with enhanced performance [20,21,22].

In contrast to conventional sensors, flexible sensors are a distinct category that can detect environmental stimuli and provide precise measurements even in challenging settings [23,24]. They exhibit superior capabilities in bending and deformation, tolerating a range of flexing and stretching. Generally, flexible sensors are composed of sensitive materials, a flexible substrate, and electrodes, requiring the materials to have good flexibility and conductivity [25,26]. The selection of suitable sensitive materials is pivotal to the sensor’s performance. Over time, an array of materials, including metal materials [27,28], carbon materials [29,30,31], two-dimensional (2D) materials [32,33], and hydrogel materials [34,35], have been pivotal in the fabrication of flexible sensors.

Despite the notable progress in flexible sensor research, the challenge of further enhancing their mechanical and sensing capabilities remains a significant hurdle [36,37]. The materials mentioned above are key factors affecting these properties. However, most materials have to make a trade-off between mechanical properties and sensing properties, and they must sacrifice part of their electrical conductivity for better flexibility and ductility, which can lead to reduced sensing properties [38,39]. A decade of research has demonstrated that composite materials can address the deficiencies of single materials, showcasing more exceptional performance. Beyond materials, the flexible also substrate plays a crucial role [40,41]. Unlike traditional sensors, the integration of flexible sensors into wearable electronic devices requires designers to choose appropriate flexible substrates and design schemes capable of withstanding substantial strain [42,43]. The primary role of the flexible substrate is to support and protect the sensitive materials. In addition to its inherent flexibility, it is essential to consider its conductivity and the interfacial adhesion to sensitive materials [44]. Typically, flexible substrates do not possess high conductivity, which might impact sensor sensitivity, yet they are vital in enhancing the linearity, detection range, and stability of flexible sensors [45,46]. The three common flexible substrates used in flexible sensors are polymers, paper, and textiles [47]. Including polyimide (PI) [48], polyester (PET), polyurethane (PU), polyvinylidene fluoride (PVDF) [49], polydimethylsiloxane (PDMS) [50], and hydrogels. However, before practical application, it is imperative to consider these materials’ performance metrics, such as thermal stability, light transmittance, electrical conductivity, chemical stability, and mechanical elasticity.

Recent market research indicates that the global flexible electronics market is anticipated to reach USD 300 billion by 2030, with a projected compound annual growth rate (CAGR) of 15%. This substantial growth is primarily driven by the escalating demand from various industries, including consumer electronics, healthcare, automotive, and industrial automation. The Asia–Pacific region is poised to capture a significant market share, propelled by rapid economic development and intensified investments in technology. Prominent players in the global flexible electronics market include Samsung, LG, BOE, and Corning. The integration of flexible electronics into these sectors is expected to catalyze new opportunities for innovation and economic growth, transforming traditional manufacturing processes and enabling the development of next-generation wearable devices, smart healthcare systems, and advanced human–computer interaction interfaces.

**Figure 1:**
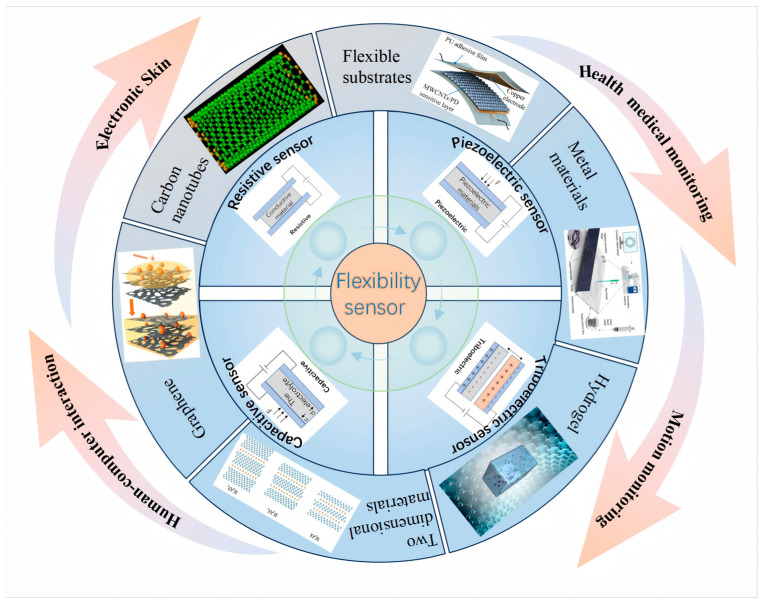
Flexible sensing technology in smart wearable devices from sensing mechanism to materials to applications.

This article reviews the trajectory of flexible sensor research and explores their expansive development prospects within the realm of smart wearable technology. The article aims to comprehensively analyze the “sensing mechanisms–materials–applications” correlation involved in flexible sensors in different application fields, as shown in Figure 1. We begin by meticulously categorizing flexible sensors based on their sensing principles, followed by an in-depth presentation of five prevalent flexible sensor materials, detailing their distinctive features, benefits, and roles in the fabrication of sensors. Leveraging this foundation, the paper distills the avant-garde applications of flexible sensors in the smart wearable sector, showcasing their remarkable potential in areas such as electronic skin, health medical monitoring, motion tracking, and human–computer interaction. Concluding the discussion, we address the current challenges confronting flexible sensors and project future trends and prospects, underscoring the pivotal role of technological innovation in propelling the evolution of flexible sensor technology.

## 2. History of Flexible Sensors

**Figure 2:**
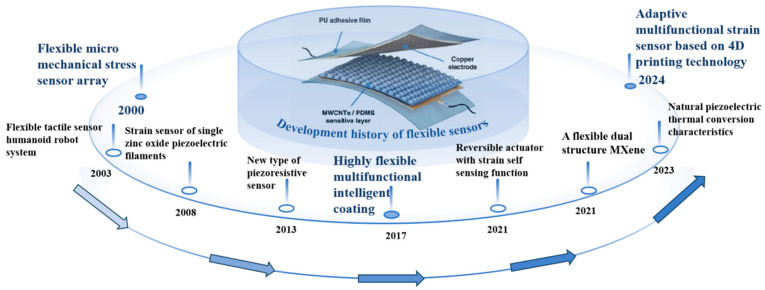
The main development process of flexible sensors in the field of intelligent wearables.

The concept of flexible sensors emerged in the 1980s, with the initial research efforts concentrated on adapting traditional sensor technologies to flexible materials. During this era, investigators primarily delved into material selection and fundamental design principles for these sensors. As the new millennium began, advancements in nanotechnology and the emergence of innovative flexible materials, such as conductive polymers and carbon nanotubes (CNTs), significantly bolstered the performance of flexible sensors. The research during this period was heavily focused on enhancing the sensors’ sensitivity, stability, and durability. Jiang et al. [51] introduced a novel microfabrication technique that played a pivotal role in the development of flexible shear stress sensor arrays, utilizing laser micromachining methods that excelled in the fabrication of nano gas-sensitive sensor arrays. This technology is characterized by its precise control, flexibility, convenience, and cost-effectiveness. In 2003, Kerpa et al. [52] proposed a tactile sensor system for flexible humanoid robots, capable of measuring object properties like hardness, pressure, and vibration through contact. Zhou et al. [53] employed a straightforward, reliable, and cost-efficient technique in 2008 to fabricate a strain sensor based on a single zinc oxide (ZnO) piezoelectric fiber, showcasing simplicity and effectiveness in sensor design. Yao et al. [54], in 2013, developed an innovative piezoresistive sensor featuring a rupture microstructure design based on graphene nanosheets wrapped around a polyurethane sponge (PUS), demonstrating ultra-high pressure sensitivity in the low-pressure range, with a minimum detectable pressure as low as 9 pascals. In 2017, Li et al. [55] successfully created a highly flexible multifunctional smart coating by dispersing multi-walled carbon nanotubes (MWCNTs) in a thermoplastic elastomer (TPE) solution, followed by an ethanol treatment process. Wang et al. [56], in 2021, developed a reversible actuator with strain self-sensing capabilities, consisting of a double-layer structure with a conductive GPLA-printed swinging mechanism and paper. Guo et al. [57] achieved significant progress in the study of conductive material MXene, designing and fabricating a double microstructure with surface microprotusions and internal hollow pores, leading to the development of a multifunctional high-performance pressure sensor with substantial impact on human health monitoring. In 2023, Xu et al. [58] introduced a flexible multimodal pulse sensor that leverages the natural piezoelectric–thermal conversion properties of human skin, combined with a thin-film temperature sensor, to measure the radial artery pulse waveform. Zhang et al. [59], in 2024, proposed an adaptive multifunctional strain sensor (AMSS) manufacturing strategy based on 4D printing technology, capitalizing on the shape memory characteristics of PLA, bio-rift structural sensing units, and the bidirectional deformation design of double-layer structures. The content above provides a concise overview of the key milestones in the evolution of flexible sensors within the smart wearable domain, as shown in Figure 2.

## 3. Sensing Mechanism and Classification of Flexible Sensors

Flexible sensors, featuring a diversity of sensing mechanisms and capable of detecting a wide array of stimuli, fundamentally transform external inputs into quantifiable electrical signals. In this section, we will delve into four prevalent sensing mechanisms, namely, flexible resistive sensors, flexible piezoelectric sensors, flexible capacitive sensors, and flexible triboelectric sensors [60]. When it comes to real applications, the choice of sensor must be tailored to the specific context. Flexible resistive sensors are noted for their pliability, straightforward fabrication, and cost-effectiveness, and they are adept at capturing subtle deformations, albeit with considerable signal drift. In contrast, flexible capacitive sensors offer a simple design with minimal signal drift but are more prone to interference from temperature and humidity fluctuations, which can compromise their stability. Furthermore, flexible triboelectric sensors stand out for their self-powered operation, eliminating the need for an external power source, and they feature a relatively simple construction with high output voltage. Regarding the scope of detection, flexible resistive and capacitive sensors are more versatile, finding applications in medical electronics, environmental monitoring, and wearable technology. However, in adversarial settings, flexible piezoelectric and triboelectric sensors come into play, offering self-sustaining power capabilities that enable extended periods of field signal monitoring and reduced costs [61]. In addition to these well-established sensing mechanisms, electrochemical flexible and stretchable sensors represent a rapidly growing area of research and development [62,63,64]. These sensors are particularly important for applications involving the detection of biochemical substances, such as glucose and lactate in sweat, saliva, and interstitial fluid. They typically operate on electrochemical principles, leveraging the chemical reactions between the electrode and the target analytes to generate measurable electrical signals. This mechanism allows for highly sensitive and specific detection, making them invaluable in health monitoring and diagnostic applications.

### 3.1. Flexible Resistive Sensors

**Figure 3:**
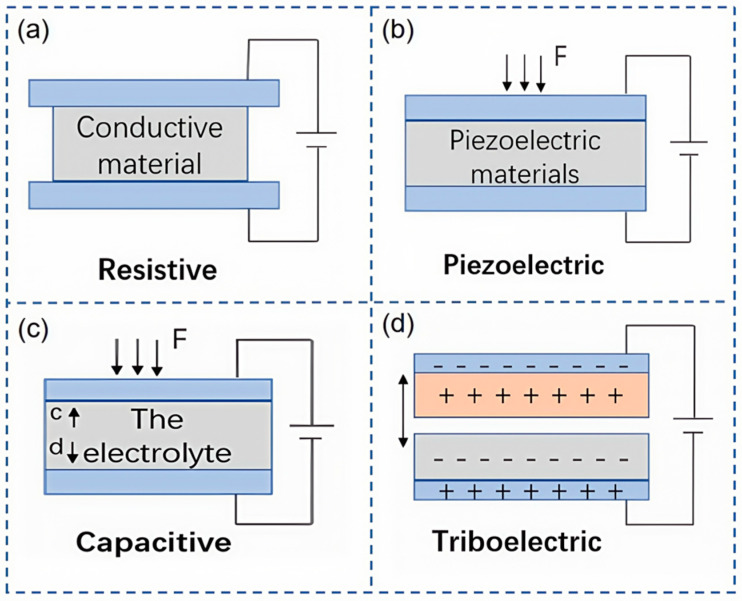
Signal conversion mechanism diagram of four flexible sensor mechanisms [65]. (**a**) Schematic diagram of flexible resistive sensor. (**b**) Schematic diagram of flexible piezoelectric sensor. (**c**) Schematic diagram of flexible capacitive sensor. (**d**) Schematic diagram of flexible triboelectric sensor.

Flexible resistive sensors are composed of an active layer, which is typically an elastic conductor or semiconductor layer that interfaces with two electrodes. The underlying principle of their operation is rooted in the piezoresistive effect, a distinctive physical phenomenon where the resistivity of semiconductor materials alters under the influence of external mechanical stress. This effect is centered on the impact of stress on the semiconductor’s band structure and the mobility of charge carriers, resulting in modifications to the resistive properties. Leveraging the piezoresistive effect, a variety of sensors have been engineered to sensitively detect variations in physical quantities such as pressure and strain, transducing these changes into electrical signals for output. Owing to their straightforward construction, reliable performance, and swift response times, flexible resistive sensors have found extensive utility across a spectrum of domains, including electronic skin, health monitoring, motion tracking, smart medical applications, and human–computer interaction. They have enhanced the accuracy and sensitivity of measurements and have driven forward the progress and evolution of associated technologies. Figure 3a illustrates the signal conversion mechanism of a flexible resistive sensor.

**Figure 4:**
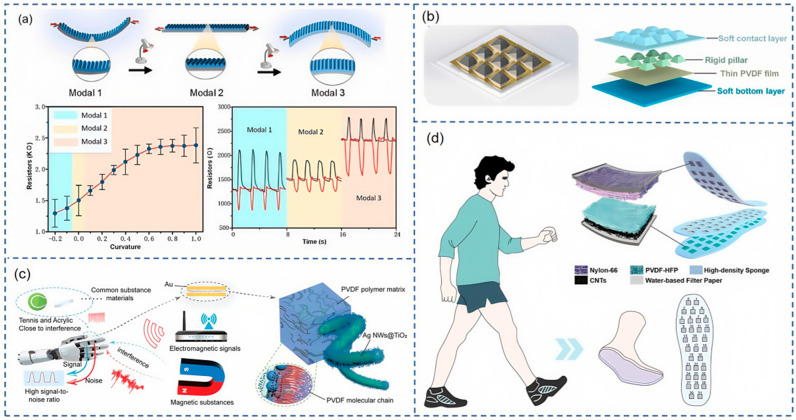
(**a**) Resistance measurement results of AMSS three modes and forward and reverse bending resistance tests [59]. (**b**) Concept design of –rigid-in-soft piezoelectric tactile sensor array [68]. (**c**) High SNR capacitive sensor based on corer–shell structure [71]. (**d**) Overview of smart insole for gait detection [74].

Zhang et al. [59] introduced a multifunctional strain sensor endowed with shape memory properties, engineered with bio-rift structural sensing units and a dual-layer structure that accommodates bidirectional deformation. This innovative sensor can discern both thermal and mechanical stimuli through the modulation of resistance signals. Under compression, the sensor’s resistance drops significantly with increasing strain, while under tension, resistance rises with strain. As shown in Figure 4a, the sensor functions in three distinct modes. Chen and colleagues [66] have developed a highly stable piezoresistive composite, employing PI fibers as the substrate and in situ polymerized polyaniline (PANI) as the semiconductor layer. This sensor demonstrates a high linear sensitivity of 35.3 kPa^−1^ within the 0.2–20 kPa pressure range, along with remarkable repeatability and dynamic stability, showing only a 3.8% signal deviation over approximately 10,000 cycles.

### 3.2. Flexible Piezoelectric Sensors

Flexible piezoelectric sensors capitalize on the piezoelectric effect, a property of certain materials that generate an electric charge in response to applied mechanical stress, thereby converting mechanical energy into electrical energy. This principle allows for the measurement of physical quantities such as pressure and vibration. Upon the application of an external force, internal polarization occurs within the piezoelectric material, resulting in the accumulation of charge on the material’s surface and the establishment of an electrical potential. When the external force is removed, the polarization process reverses, and the charges dissipate. The magnitude of the current generated can be used to determine the intensity of the applied force. The signal transduction mechanism of the flexible piezoelectric sensor is shown in Figure 3b.

Upon the application of external force along a defined axis, certain dielectric materials experience deformation, accompanied by internal polarization. This polarization leads to the emergence of positive and negative charges on the opposing faces of the dielectric, with the magnitude of these charges being directly proportional to the force applied. When the external force is withdrawn, the dielectric reverts to its original, neutral state, completing the cycle of the reversible direct piezoelectric effect. In an inverse process, the application of an electric field to piezoelectric materials triggers mechanical deformation along a specific axis, proportional to the electric field’s intensity, a phenomenon referred to as the converse piezoelectric effect. Piezoelectric sensors, renowned for their self-sustaining nature without the need for an external power source, excel in the detection of both dynamic and static forces, particularly in capturing rapidly fluctuating forces. This capability has led to their extensive use in a variety of measurement environments.

Capitalizing on the characteristics of piezoelectric materials, there is a growing trend toward developing biosignal monitoring systems that are compatible with human skin, manifesting in the form of piezoelectric sensors. Li et al. [67] employed 3D printing to fabricate a continuous double-layered lead zirconate titanate (PZT) ceramic scaffold, which, when integrated with epoxy resin and interdigital electrodes, formed a multifunctional device. As shown in Figure 4b, Huang et al. [68] have introduced an innovative rigid-in-soft structure, characterized by a truncated pyramid shape and a soft bottom layer, which significantly enhances the efficiency of force transmission. This sensor exhibits exceptional flexibility and reliability, positioning it as a highly promising solution for the detection of dynamic stimuli in robotics applications, including slip detection and vibration monitoring.

### 3.3. Flexible Capacitive Sensors

Flexible capacitive sensors, crafted from flexible materials, merge the principles of capacitive sensing technology with the inherent properties of flexibility. They are renowned for their exceptional pliability and extensibility [69,70]. Typically, a flexible capacitive sensor is composed of two parallel plate electrodes with a dielectric layer sandwiched between them. Upon the application of external forces, the thickness or area of the dielectric layer is altered, consequently affecting the capacitance, as delineated by Equation (1):

(1) $$C=\varepsilon \times \frac{S}{d}$$

where *C* is the capacitance of the capacitor, *S* is the area of the electrode, *ε* is the dielectric constant of the dielectric between the electrodes, and *d* is the distance between the electrodes.

In the absence of any object near the sensor, the capacitance between the electrodes maintains a relatively stable value. This stability arises because the distance, area, and dielectric constant between the electrodes remain unchanged. However, when an object—such as a finger, liquid, or any conductive or non-conductive material—approaches or touches the sensor, it disrupts the equilibrium by altering either *ε* or *d*, resulting in a change in capacitance. With conductive objects, they may effectively integrate with the electrodes, thus modifying *S* or *d*. Conversely, non-conductive objects primarily affect *ε*.

Figure 3c depicts the signal transduction mechanism of a flexible capacitive sensor. Within the domain of smart wearable devices, flexible capacitive sensors are highly regarded for their exceptional flexibility, stability, sensitivity, and straightforward construction. These attributes have piqued the interest of numerous researchers, leading to the conception of innovative and daring ideas. As shown in Figure 4c, Han et al. [71] proposed a highly interference-resistant flexible capacitive pressure sensor with a dielectric layer made of PVDF, silver nanowires (AgNWs), and titanium dioxide (TiO_2_) films, which maintains a high signal-to-noise ratio even in the presence of various interference sources. Integrated with a smart glove sensing system, it offers a novel perspective for the development of highly interference-resistant flexible capacitive pressure sensors. Inspired by the Miura-ori structure, Sun et al. [72] developed a symmetric Miura-ori capacitive sensor (SMC) designed to establish a positive correlation between sensitivity and pressure, adjustable by modifying the SMC sensor’s structure within a tunable pressure range. The capacitance is determined by the synergistic effect of the distance and relative area between electrodes that align with the Miura-ori structure. This sensor is capable of monitoring general physiological signals under conditions of high stretchability and high environmental adaptability.

### 3.4. Flexible Triboelectric Sensors

Flexible triboelectric sensors operate on the principles of the triboelectric effect and electrostatic induction. When two materials with opposite polarities come into contact, their surfaces generate static charges of opposite signs. As these materials separate, electrostatic induction on the electrodes positioned on the reverse side leads to the generation of induced charges, creating a potential difference. This potential difference propels the flow of electrons through an external circuit, continuing until the two materials are fully separated and an equilibrium state is achieved [73]. Through this process, flexible triboelectric sensors are capable of transforming external mechanical forces or movements into electrical signals for output. This mechanism enables flexible triboelectric sensors to produce signals during both the application and release of pressure, as shown in Figure 3d. Triboelectric sensors are available in various configurations, including contact-separation, sliding, and pressure types. While these sensors differ slightly in structure and working principles, they all fundamentally rely on variations in electrostatic induction and charge distribution.

Contact-separation triboelectric sensors function by harnessing the charge transfer that occurs between two distinct materials during the processes of contact and separation. Upon contact, due to their varying electron affinity, these materials exchange charges, leading to one material acquiring a positive charge while the other becomes negatively charged. As the materials separate, electrostatic induction generates induced charges on the electrodes, thereby producing an electrical signal. These sensors boast a simple structure, facilitating ease of manufacturing and integration. Their sensitivity to contact and separation actions renders them ideal for applications that necessitate the detection of object contact or separation states.

Sliding triboelectric sensors harness the triboelectric effect that arises from the relative motion of two materials in contact. As these materials slide against each other, friction at the interface leads to the transfer of charges, which then results in the generation of an electrical signal on the electrodes. These sensors are particularly sensitive to the act of sliding and are adept at capturing details such as the velocity, direction, and distance of an object’s movement.

Pressure-based triboelectric sensors operate on the principle of material deformation and the consequent triboelectric effect induced by the application of pressure. Upon the exertion of pressure, the material deforms and comes into contact with another material, resulting in the generation of triboelectric charges, which, in turn, produce an electrical signal at the electrodes. The amplitude of this electrical signal is directly proportional to the magnitude of the applied pressure. These sensors exhibit sensitivity to pressure fluctuations and are adept at detecting the distribution and variation of pressure exerted by objects.

As shown in Figure 4d, Zhou et al. [74] have engineered a plantar pressure sensor array and gait analysis system utilizing a flexible triboelectric pressure sensor (FTPS) array. This innovative system delivers high sensitivity of 45.1 mV kPa^−1^ within the 40–200 kPa range and maintains a high degree of precision, reaching 19.4 mV kPa^−1^ in the 200–400 kPa range. Venkatesan et al. [73] developed a triboelectric nanogenerator (TENG) by employing a PVDF-carbon fiber (P-CF) nanocomposite as the negative triboelectric layer and a TPU nonwoven fabric as the positive triboelectric layer. The resulting TENG device has been successfully utilized for real-time healthcare monitoring (HCM) and in the context of polysomnography (PSG) studies.

## 4. Commonly Used Materials for Flexible Sensors

### 4.1. Flexible Substrates

Flexible substrates, or flexible printed circuit boards (FPCs), represent a class of printed circuit boards that are celebrated for their remarkable reliability and flexibility. These substrates, with their lightweight, thin design, and capacity for free bending and folding, offer unparalleled flexibility in applications where space is at a premium. They are an integral component within electronic devices, adept at fulfilling a spectrum of sophisticated and varied demands. The materials commonly employed for these substrates encompass PI, PET, PDMS, polyether ether ketone (PEEK), PVDF, and textile materials, each bringing unique properties to the table.

Recent market research indicates that the global flexible substrate market is anticipated to reach USD 28.7 billion by 2031, with a projected compound annual growth rate (CAGR) of 18.9%. This growth is primarily driven by the escalating demand from the electronics sector, especially for applications such as flexible printed circuit boards and smart solar modules. The Asia–Pacific region is poised to capture a substantial market share, propelled by rapid economic growth and intensified investments in the electronics industry. Key players dominating the global flexible substrate market include DuPont Teijin Films, Schott, Corning, and Kolon Industries.

**Figure 5:**
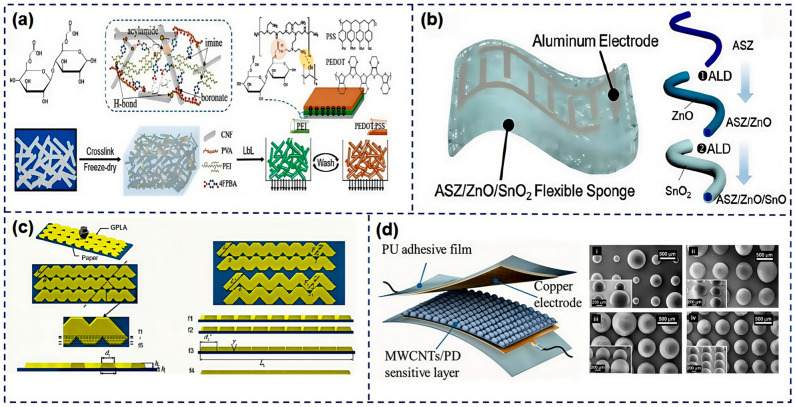
(**a**) Schematic of flexible CNF porous material preparation and illustration of LbL self-assembly with PEI and PEDOT: PSS [75]. (**b**) Fabrication and structure schematic of flexible resistive gas sensors [76]. (**c**) Four-dimensional printing methods and schematic diagram of the sensor-reversible actuator [56]. (**d**) Schematic diagram of the sensor with the MWCNT/PDMS sensitive film [58].

The development and application of flexible electrodes have consistently been a focal point of research. As shown in Figure 5a, Du et al. [75] crafted a three-dimensional, porous flexible substrate by alternately depositing positively charged polyethyleneimine (PEI) and negatively charged conductive polymer, poly:poly, onto cellulose nanofiber (CNF) porous scaffolds, employing a pressure gradient-assisted layer-by-layer self-assembly technique. As shown in Figure 5b, Li et al. [76] introduced, for the first time, a novel wearable gas sensor based on entirely inorganic ASZ (Al_2_O_3_-stabilized ZrO_2_)/ZnO/SnO_2_ nanofibers. The flexible ASZ ceramic sponge substrate, with a Young’s modulus of 4.15 MPa, and the ultra-thin ZnO/SnO_2_ sensing layer confer upon the wearable gas sensor promising attributes such as ultra-flexibility (with a bending radius of 5 mm), high air permeability, and low weight. Zhang et al. [59] utilized polylactic acid (PLA) as the material for the flexible substrate, achieving shape memory characteristics and programmable sensing properties through 4D printing technology. In this research, PLA layers were printed onto pre-strained elastic band layers, creating a double-layer structure. This flexible substrate not only provides flexible support but also integrates closely with the sensor’s functionality through its material properties, such as shape memory, enabling programmable control over sensing performance. Common copy paper, with a thickness of 0.7 mm, was used as the base material by Wang et al. [56], on which conductive graphene polylactic acid (GPLA) material was printed using fused deposition modeling 3D printing technology, forming a double-layer structure. As shown in Figure 5c, the micro-fiber structure on the surface of the copy paper allows for a strong bond with the printed conductive graphene polylactic acid material. This structure leverages the shape memory effect of GPLA, which can be programmed to control deformation at specific temperatures, combined with their specially designed “swing path” structure, enabling changes in contact resistance during longitudinal deformation, thus achieving strain self-sensing functionality. As shown in Figure 5d, Xu et al. [58] employed high-precision equipment to prepare templates with multi-level microstructures through projection micro stereo lithography (PμSL) 3D printing technology then cast TPU solution onto the printed templates and, after curing, peeled it off to obtain TPU molds with inverse microstructures. Subsequently, the prepared TPU molds were reversed and cast with MWCNT/PDMS conductive solutions. Finally, the TPU films coated with conductive solutions were heated and cured and then peeled off from the templates to form a sensitive layer with micro-hierarchical structures. This TPU substrate, with its excellent flexibility and elasticity, is highly suitable for manufacturing pressure sensors capable of detecting subtle pressure changes. Tian et al. [77] proposed a flexible sensor for continuous blood pressure monitoring suitable for wearable use, with a substrate made of PI as the base material. Two concentric Cr/Pt film rings were deposited on the flexible substrate as thermosensitive elements. The flexible substrate closely conforms to the skin surface, utilizing the skin as a natural piezothermoelectric conversion material. On the back of the flexible substrate, a porous silver particle-enhanced PDMS layer was integrated, which not only improved the sensor’s piezothermoelectric conversion efficiency but also served to secure the sensor on the wrist.

### 4.2. Metal Materials

Metal materials are renowned for their exceptional electrical conductivity and their ability to substantially fulfill the demands for flexibility and ductility in flexible sensors. As nanotechnology progresses, the utilization of nanoparticles and nanowires in flexible sensor applications has expanded significantly. These nanostructures offer not only superb electrical conductivity but also a marked improvement in the ductility of metallic materials, which is well-suited to the requirements of flexible sensors. Owing to their high conductivity, stability, and reliability, metal materials have emerged as the ideal choice for the construction of flexible sensors.

**Figure 6:**
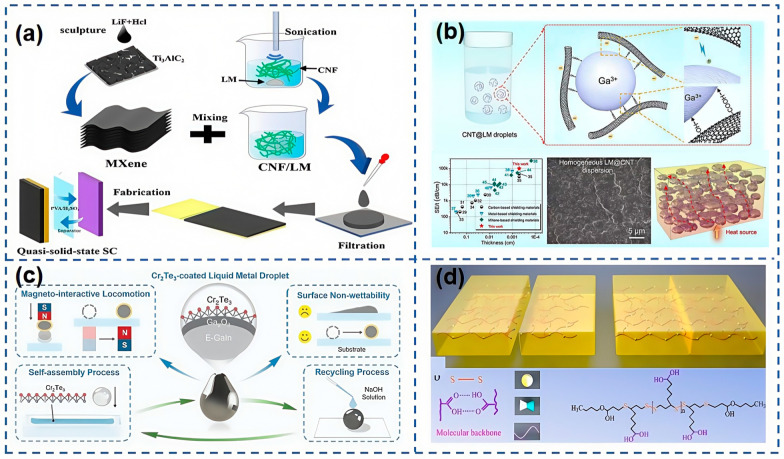
(**a**) Schematic illustration of the fabrication process for MXene/CNF/GI composite film and supercapacitor [78]. (**b**) Schematic illustration of the interface interaction of the CNT@LM droplets [79]. (**c**) Schematic illustration of the fabrication process and the structure of CT-LM droplets with its surface non-wettability, recycling ability, and easy magnetointeractive locomotion [80]. (**d**) Schematic diagram of fracture and instant healing of α-thioctic acid–butyl acrylate copolymer by hydrogen bond and dynamic disulfide bond [84].

As shown in Figure 6a, Yuan et al. [78] developed a composite film made from a blend of liquid metal (LM), MXene, and CNF, resulting in a porous architecture that boasts enhanced electrical conductivity. This improvement facilitates the transport of ions and electrons within the material, thereby conferring superior electrochemical performance. As shown in Figure 6b, Yi et al. [79] synthesized highly stable LM droplets by reacting carboxylated CNTs with Ga^3+^ ions, showcasing excellent dispersion stability with no significant sedimentation even after 30 days. Li et al. [80] introduce an innovative strategy that combines liquid metals with two-dimensional magnetic materials, resulting in the formation of chromium telluride-coated liquid metal (CT–LM) droplets through a straightforward self-assembly technique. These CT–LM droplets showcase an ability to undergo controlled deformation and movement in response to magnetic fields, exhibit non-stick properties to a variety of surfaces, and facilitate the cost-effective recycling of materials, as shown in Figure 6c. Peng et al. [81] demonstrated the formation of nanodroplets from CNTs and LM under ultrasonication, which can serve as initiators for the radical in situ polymerization of water-soluble vinyl monomers at room temperature, eliminating the need for additional initiators. Their findings indicated that these nanodroplets could act as both initiators and crosslinkers in the synthesis of hydrogels, enabling the swift fabrication of multifunctional hydrogels. These hydrogels, capable of patterning and rapid injection molding, hold extensive potential for applications in flexible wearable devices.

LM ink stands out as an ideal conductive element for the 3D printing of smart electronic garments. Wu et al. [82] introduced an LM ink capable of directly printing functional circuits onto commercial attire, thereby crafting smart wearables that integrate a spectrum of functionalities, including tactile sensing, motion tracking, and human–computer interaction. In the realm of wearable technology, flexible composite materials embedded with magnetic fillers [83] not only demonstrate enhanced conductivity upon stretching but also showcase self-healing properties. As shown in Figure 6d, Wang et al. [84] employed a bio-based copolymer of α-thioacid and butyl acrylate (BA), known for its recyclability, self-healing capabilities, and rich network of hydrogen and disulfide bonds, as the substrate material. By fusing the superior fluidity of LM with the outstanding self-healing attributes of this flexible base material, they achieved instantaneous self-healing, resulting in flexible devices with real-time mechanical and electrical self-healing properties. This innovation presents a novel pathway for the development of smart wearable devices.

### 4.3. Carbon Material

Carbon materials typically utilized in flexible wearable electronic sensors encompass CNTs, graphene, and carbon fibers. These carbon-based materials are renowned for their ability to withstand deformations such as bending, folding, compressing, or stretching without breaking or experiencing a marked decrease in performance. They exhibit excellent electrical conductivity, superior flexibility, and maintain stable properties across diverse environmental conditions. Such attributes have positioned them as vital components in a spectrum of applications, from smart wearable devices and energy storage to biomedical applications and information technology, showcasing a vast potential for future utilization.

**Figure 7:**
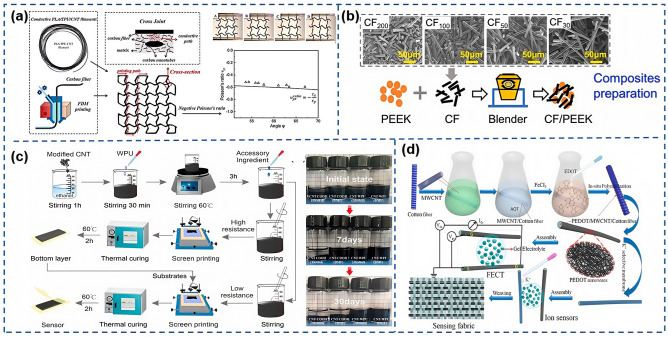
(**a**) Printing and tensile failure process of printed negative Poisson’s ratio CFRSMCs [85]. (**b**) The preparation process of CF/PEEK powder composites [86]. (**c**) Fabrication process of the sensor and the solubility of MWCNTs-COOH and g-MWCNTs in screen-printing ink with ethanol and DMF solvents in different time periods: initial state; 7 days; 30 days [88]. (**d**) Schematic illustration of the fabrication of MWCNT functionalized PEDOT nanowires and its applications in K^+^ sensors based on fiber organic electrochemical transistors [90].

As shown in Figure 7a, Dong et al. [85] employed 4D printing to craft continuous carbon fiber-reinforced shape memory composites (CFRSMCs) with electro-induced shape memory properties. This process involved the initial fabrication of conductive filaments from a PLA/TPU/carbon nanotube hybrid nanocomposite, followed by the integration of these filaments with continuous carbon fibers through advanced 3D printing techniques, yielding CFRSMCs with varying carbon fiber concentrations. These composites showcase exceptional energy storage modulus, rigidity, and temperature responsiveness, making them well-suited for a range of applications, including deployable trusses, adaptive energy absorption systems, and large-scale orthopedic materials. As shown in Figure 7b, Lu et al. [86] utilized PEEK powder as a matrix material and carbon fibers as the reinforcing phase, with a fiber diameter of 7 μm and lengths between 63 to 342 μm. By mechanically blending dried PEEK powder with carbon fibers and leveraging a custom screw extrusion 3D printer, they produced composites that exhibit excellent biocompatibility and corrosion resistance, ideal for the fabrication of artificial joints and fracture fixation plates to facilitate patient mobility. Pang et al. [87] achieved the plasticization of solid graphene oxide (GO) through water intercalation, conferring the necessary plasticity for foaming, and then generated foam through the use of reactive gases or chemical reagents. The hydroplastic foaming of graphene aerogels presents extensive application prospects in advanced composite materials and smart electronics. As shown in Figure 7c, Xia et al. [88] incorporated modified multi-walled CNTs into aqueous PU and employed screen printing to fabricate a double-crack structured sensor capable of detecting subtle tensions like pulses and sounds, as well as deciphering Morse code through minute finger movements, aiding communication for Parkinson’s patients. Furthermore, it can detect large strain movements such as knee joint motion and, when combined with algorithms, identify human activities to assess the risk of falls in the elderly based on motion trends. Wang et al. [89] introduced a novel flexible sensor based on carbon nanotube paper films (CNTF) and stress-induced conical structures (SSFS), capable of sensing a variety of human signals, including pulse, vocal cord vibrations, wrist flexion, and foot pressure, and can also be integrated into car tires for vehicle status monitoring. This flexible sensor, with its superior detection capabilities, demonstrates immense potential in electronic skin, human–computer interaction, and health monitoring. As shown in Figure 7d, Wang et al. [90] successfully merged organic electrochemical transistors (FECTs) technology with poly (3, 4-ethylenedioxythiophene)/multi-walled carbon nanotubes (PEDOT/MWCNT) composite sensing channels to develop a novel flexible potassium ion sensor. This innovative design significantly enhanced the morphology of PEDOT on the optical fiber substrate, transforming it from a disordered dispersion to a tightly interconnected, web-like conductive structure, greatly improving the electrical performance of FECTs. Moreover, the sensor’s excellent integrability allows for easy embedding into fabrics through manual weaving, enhancing its portability and comfort and laying a solid foundation for its widespread application in future wearable devices.

### 4.4. Two-Dimensional Materials

**Figure 8:**
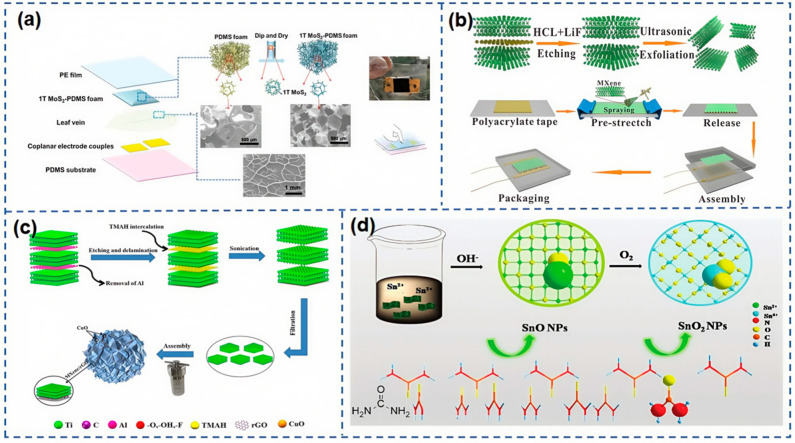
(**a**) Fabrication process and structure of the 1T MoS_2_-PDMS foam pressure sensor [92]. (**b**) The procedure diagram of preparation procedure of the piezoresistive sensor based on MXene composite with wrinkle structure [95]. (**c**) Schematic illustration of fabrication process of 3D MXene/rGO/CuO aerogel [96]. (**d**) Schematic of the chemical reactions during the preparation of SnO-SnO_2_/Ti_3_C_2_T_x_ nanocomposites [97].

Two-dimensional materials represent a class of distinctive nanomaterials, celebrated for their unique attributes, including a large surface area to volume ratio, exceptional mechanical strength, electrical conductivity, and distinctive electronic and optical properties. These characteristics render them an ideal choice for conductive sensitive materials in piezoresistive sensors. Prominent examples of 2D materials include boron nitride (BN), molybdenum disulfide (MoS_2_), tungsten disulfide (WS_2_), molybdenum diselenide (MoSe_2_), tungsten diselenide (WSe_2_), and MXene materials. As research into 2D materials deepens, these materials with their unique properties are increasingly becoming a focal point of interest in the realms of material science and nanotechnology. Alwin et al. [91] developed flexible monolayer MoS_2_ temperature sensors and arrays that can detect temperature changes within microseconds, a speed over 100 times faster than that of flexible thin-film metal sensors. These sensors also possess a high resistive temperature coefficient of about 1–2%/K and demonstrate stable operation in cyclic and long-term measurements when encapsulated with aluminum oxide. These findings, combined with their biocompatibility, position these devices as prime candidates for biomedical sensor arrays and a myriad of other Internet of things applications. As shown in Figure 8a, Yang et al. [92] fabricated high-performance pressure sensors utilizing 1T-MoS_2_ within PDMS foam as the conductive active layer and layered microporous channels as the spacer. Owing to its superior conductivity and innovative structural design, the 1T-MoS_2_-PDMS outperforms previously reported devices. It boasts a high sensitivity of 1036.04 KPa across a broad linear range from 1 to 23 KPa, a swift response time of less than 50 ms, an ultra-low detection pressure threshold of 6.2Pa, and remarkable stability in repetitive loading and unloading cycles.

MXene, initially discovered in 2011, has emerged as a novel class of 2D materials, consisting of a few atomic layers of transition metal carbides, nitrides, or carbonitrides [93]. This material is characterized by its near-metallic electrical conductivity and superior mechanical properties, which align well with the demands for sensing and mechanical performance in flexible sensors. Furthermore, the presence of hydroxyl or fluorine–oxygen groups on its surface endows MXene with exceptional hydrophilicity, aiding in the formation of stable colloidal solutions. In addition to its electromagnetic shielding capabilities, which are ideal for developing lightweight and efficient shielding materials, MXene also exhibits promising biocompatibility and drug-loading properties, positioning it as a candidate for biomedical applications [94]. Given these attributes, MXene, as a rising star among 2D materials, is poised to have extensive applications in flexible sensors and is anticipated to assume a more significant role in forthcoming technologies.

To expand the detection capabilities of MXene-based flexible sensors, it is crucial to reduce the interactions between MXene flakes and to engineer new conductive networks. As shown in Figure 8b, Yan et al. [95] integrated 2D MXene materials with a wrinkled structure to craft a flexible, highly sensitive piezoresistive sensor. By spraying the active material onto the surface of a pre-stretched polyacrylate strip, they fabricated a MXene composite sensor with a wrinkled structure. This approach is straightforward, efficient, and cost-effective. The sensor, based on MXene composites, exhibits exceptional sensitivity (148.26 kPa^−1^), a wide pressure range (up to 16 kPa), a rapid response time (120 ms), and superb durability (over 13,000 cycles). Moreover, with its extraordinary sensing performance and flexibility, the sensor can detect human physiological signals, monitor the posture of intelligent robots, and chart spatial pressure distributions, indicating substantial potential in physiological analysis systems, humanoid robots, and biomedical prosthetics. As shown in Figure 8c, Liu et al. [96] introduced an ion-conductive composite film made of reduced graphene oxide (rGO), nitrogen-doped MXene Ti_3_C_2_T_x_ (N-MXene), and TiO_2_, capable of sensing 4–40 ppm formaldehyde vapor at room temperature (20 °C) and ambient humidity. Under various humidity conditions near 4 ppm HCHO, the ternary sensor achieved an average reversible response of 26% at 54% relative humidity (RH). It also demonstrated excellent repeatability, long-term stability, and selectivity. As shown in Figure 8d, Wang et al. [97], utilizing Ti_3_C_2_T_x_ as a titanium source, successfully prepared well-defined Ti_3_C_2_T_x_-TiO_2_ nanocomposites through a straightforward one-step hydrothermal synthesis method. Thanks to the formation of interfacial heterojunctions and the modulation of carrier density, the room-temperature detection response of the Ti_3_C_2_T_x_-TiO_2_ sensor to various volatile organic compounds (VOCs) is 1.5 to 12.6 times higher than that of pure MXene sensors. Additionally, the nanocomposite sensor shows a more pronounced response to hexanal (the gas response of the Ti_3_C_2_T_x_-TiO_2_ sensor to 10 ppm hexanal is approximately 3.4%).

### 4.5. Hydrogel

Hydrogels are highly hydrophilic three-dimensional networked gels that can quickly swell in aqueous solutions while retaining substantial amounts of water without dissolving. Additionally, they exhibit certain mechanical and electrical properties, making them particularly advantageous for the fabrication of flexible sensors.

**Figure 9:**
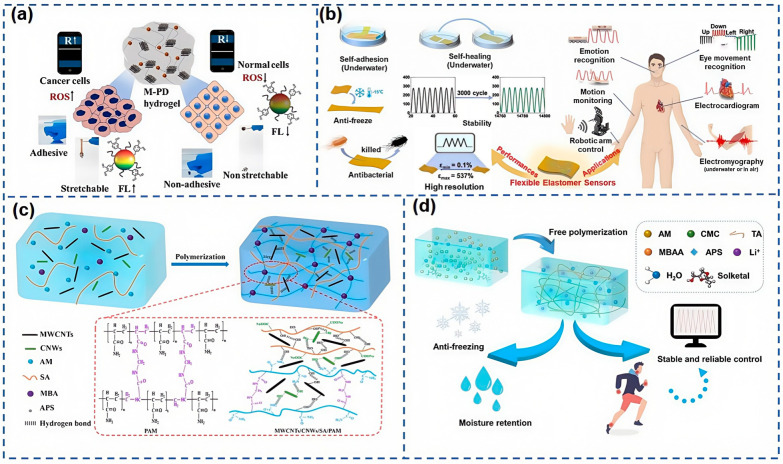
(**a**) Simple strategy diagram of cancer cell mechanical and electronic regulation hydrogel based on MXene immobilized hyaluronic acid polymer dots (M-PD) [98]. (**b**) The multifunctionality of elastomers and their application in flexible sensing [99]. (**c**) Schematic structure of MWCNTs/CNMs/PAM/SA hydrogels [101]. (**d**) Schematic diagram of the manufacturing process and advantages of PTSL hydrogel [102].

As shown in Figure 9a, Dey et al. [98] have crafted a mechanically and electronically adjustable hydrogel based on MXene immobilized hyaluronic acid polymer dots (M-PD) for the modulation of cancer cells. This flexible M-PD sensor, characterized by its high sensitivity and low detection threshold, can be integrated with a wireless device on a smartphone, facilitating real-time monitoring of cancer. As shown in Figure 9b, Li et al. [99] developed a low-modulus gelatinous elastomer through a Diels–Alder (DA) reaction, achieving ultra-fast self-healing at room temperature via molecular chain entanglement. The conductive elastomer-based flexible sensor exhibits a low modulus (6.41 kPa) and rapid self-healing properties, combining the high durability and environmental stability of an elastomer with the benefits of being highly recyclable and reusable. It features a wide detection range, swift response times, freeze resistance, antimicrobial properties, and excellent biocompatibility. Huang et al. [100] introduced a dual physically crosslinked polyacrylamide (PAM)/sodium hyaluronate (HA)/montmorillonite (MMT) hydrogel, synthesized through a straightforward strategy, showcasing exceptional stretchability, high tensile stress, high toughness, and minimal hysteresis. Flexible strain sensors made from this hydrogel demonstrate quick response and recovery times, along with superior stability over 500 cycles, making them suitable for applications in motion detection and gesture recognition. The aforementioned MXene, a 2D material, can be combined with polyacrylic acid (PAA), carboxymethyl cellulose potassium (SCMC), and metal ions (Ni^2+^, Al^3+^, or Sn^0.4+^) to form MXene-based conductive hydrogels. These hydrogels offer excellent mechanical properties and self-healing capabilities, as well as good electrical conductivity and adhesion, enabling flexible sensors based on them to effectively detect human motion and electrocardiograms, and hold broad application prospects in the realm of flexible electronics. Conductive hydrogels have been extensively applied in the fields of human–computer interaction and motion detection. As shown in Figure 9c, Feng et al. [101] constructed a double crosslinked network composite hydrogel, filling it with MWCNTs to significantly enhance the hydrogel’s electrical conductivity, thus making it an effective flexible tactile sensor capable of recognizing various gesture movements and temperature changes in electrical signals. As shown in Figure 9d, Cui et al. [102] developed a tannin (TA)-reinforced dual-network hydrogel based on polyacrylamide (PAM) and sodium carboxymethyl cellulose (CMC), incorporating green solvent Solketal and lithium chloride (LiCl) to provide more modification possibilities for the hydrogel, enhancing water content and mechanical performance consistency at 30–90% RH. This composite hydrogel (PTSL) boasts long-term stability, excellent mechanical strength, and freeze resistance.

Hydrogels are emerging as an exemplary material for flexible sensors, with extensive potential in the realms of smart wearable technology and biomedicine. They offer the ability to embed conductive materials like CNTs within their structure, resulting in the creation of highly sensitive flexible strain sensors. Furthermore, the combination of hydrogels with 2D materials such as MXene opens up avenues for monitoring human movement and advancing intelligent medical applications. As research progresses, the performance of hydrogels is being progressively uncovered and optimized, contributing significantly to the enhancement of human health and the conveniences of daily living.

### 4.6. Summary

This section explores the various materials used in flexible sensors and their research advancements, encompassing a range of materials, including flexible substrates, metallic materials, carbon materials, 2D materials, and hydrogels.

First, flexible substrates serve as the foundational support for flexible sensors, characterized by their lightweight, thin-walled, and easily bendable properties, which enhance the adaptability of electronic devices in confined spaces. Second, metallic materials play a crucial role in flexible sensors due to their high electrical conductivity, stability, and reliability. Advances in nanotechnology have facilitated the increasing use of nanoparticles and nanowires in flexible sensors, further enhancing their performance. Carbon materials are vital in flexible sensors owing to their excellent electrical conductivity and flexibility. These materials can undergo bending, folding, and other deformations while maintaining stability across various environmental conditions, showcasing a wide range of application potential. Two-dimensional materials, such as hexagonal BN, MoS_2_, and MXene, are ideal candidates for piezoresistive sensors due to their unique electrical, optical, and mechanical properties. Hydrogels, which are highly hydrophilic three-dimensional networked gels, offer significant advantages in the fabrication of flexible sensors. Their high water content, favorable mechanical properties, and electrical performance make them increasingly popular in flexible sensor applications. By embedding conductive materials within hydrogels, it is possible to create highly sensitive flexible strain sensors for monitoring human motion, electrocardiograms, and more.

In summary, as material science continues to advance and technology, the performance of flexible sensors will be continuously enhanced, and their application fields will be further expanded. Currently, the compounding of these individual materials to create composite materials that harness the advantages of various materials has become an important approach to fabricating high-performance flexible sensors. As shown in Table 1, combining the sensing mechanism classification from Chapter 3 and the material selection from Chapter 4, this chapter provides a comprehensive summary of the materials used in flexible sensors, including flexible piezoresistive sensors, flexible capacitive sensors, and flexible triboelectric sensors. It also outlines the key performance indicators—such as the detection range, sensitivity, and cyclic stability—of sensors prepared from different composite materials across these three categories.

## 5. Application of Flexible Sensors

Flexible sensors, celebrated for their distinctive pliability, stretchability, and adaptability, are finding extensive applications in a multitude of domains, including electronic skin, health monitoring, motion tracking, smart healthcare, and human–computer interaction. By being integrated into garments or directly onto the skin, these sensors can monitor health status and physical activities, thereby significantly elevating our quality of life and introducing a myriad of conveniences and innovations into our everyday existence.

### 5.1. Electronic Skin

Electronic skin represents a groundbreaking innovation in the realm of flexible, wearable sensors, distinguished by its exceptional flexibility, transparency, and biocompatibility. It emulates the sensory capabilities of human skin, providing a nuanced perception of the surrounding environment. With the ability to bend and stretch just like our own skin, it can seamlessly conform to the contours of the human body or robotic limbs. This technology transcends the limitations of traditional sensors, which are often rigid and prone to damage. The superior attributes of electronic skin enable it to effortlessly detect signals from surfaces of complex geometries. As the wearable technology sector advances towards the integration of electronic skin, it confronts the ongoing challenge of seamlessly integrating flexible sensors with human skin. In response to this challenge, Dai [111] and his team have engineered an adhesive electronic skin that can securely adhere to the human skin, designed for the sensitive detection of mechanical stimuli. Bandodkar et al. developed a fully printed temporary tattoo glucose sensor for non-invasive monitoring of blood sugar levels. The sensor combines reverse ionophoresis and enzyme–amperometric biosensing technology to respond linearly to physiological glucose levels with minimal interference. Human trials have shown that it can effectively monitor changes in blood sugar and correlates with commercial glucose meter results, showing potential in diabetes management and monitoring other physiological analytes [112,113].

**Figure 10:**
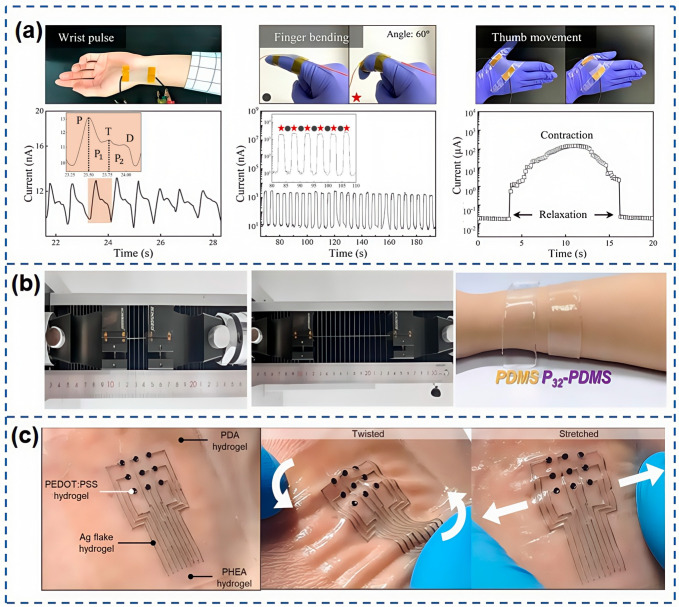
(**a**) Pulse waveform measured on the left wrist; periodic signal transmission of index finger bending motion; signal transmission for controlling thumb movement [114]. (**b**) Photos comparing the adhesion of PDMS and P32-PDMS elastomers on the arm skin in their original and stretched states [115]. (**c**) The manufactured electronic skin patch is attached to the skin. Electronic skin patches adhere closely to the skin under different conditions of twisting and stretching [116].

Gu et al. [114] have crafted an innovative neuromorphic polyurethane (NP-PU) matrix, inspired by the intricate network of metal microdendrites and nanostars, which serves as the foundation for the fabrication of highly sensitive piezoresistive pressure sensors. These sensors boast an impressive sensitivity of 160.3 kPa^−1^ and an exceptionally low detection resolution limit of 4 Pa, making them ideal for applications in medical electronic skin. As shown in Figure 10a, the sensor, when affixed to the wrist, can meticulously monitor the micro-pressure of the radial artery, offering critical insights for the diagnosis of vascular sclerosis. Moreover, the same figure illustrates the sensor’s ability to detect movements in hand joints and muscles, translating hand gestures into electrical signals with precision. Cai et al. [115] have engineered a flexible sensor with a sandwich structure, employing AgNWs as conductive fillers and P32-PDMS elastomer as a pliant substrate. This sensor exhibits commendable sensitivity (GF = 17.27), an expansive detection range spanning 0–100% strain, and remarkable cyclic stability through 5000 cycles. The P32-PDMS elastomer, as shown in Figure 10b, possesses excellent elongation at break and flexibility, rendering it an apt choice for flexible sensors. Unlike PDMS elastomers, P32-PDMS elastomers adhere well to human skin, ensuring the sensor remains in place. This electronic skin is adept at detecting a spectrum of human movements, generating reliable signals for both subtle internal activities like pulse and voice, as well as more pronounced actions such as pressing, finger bending, and elbow flexion. Shin et al. [116] have developed a functional hydrogel-based electronic skin patch designed for the accelerated healing and monitoring of skin wounds. This hydrogel closely mimics the properties of human tissue in terms of water content and Young’s modulus, facilitating a stable interface with tissue in fluctuating physiological settings. As shown in Figure 10c, PEDOT:PSS hydrogel, silver flake hydrogel, and PDA hydrogel are utilized as working electrodes, interconnects, and tissue adhesives, while PHEA hydrogel and PVA hydrogel form the matrix and encapsulation. This electronic skin patch seamlessly integrates with the skin, maintaining superior functional stability even under conditions of twisting and stretching, and ensuring consistent contact with the skin. The patch facilitates wound healing through effective fibroblast migration, proliferation, and differentiation via EF stimulation and iontophoretic drug delivery, while also providing real-time monitoring of the healing process through impedance mapping. These features underscore the multifaceted potential of hydrogel-based bioelectronics in intelligent medical applications.

### 5.2. Health Medical Monitoring

Flexible sensors, renowned for their snug fit, non-invasive monitoring capabilities, and dependable performance, have become integral to healthcare monitoring systems. These systems empower individuals to stay abreast of their health status at all times, facilitating the early identification of potential health concerns and the prompt initiation of necessary treatment protocols. Within the domain of wearable technology, these sensors play a pivotal role by continuously monitoring critical physiological metrics such as heart rate, blood pressure, and respiratory rate. For example, Han et al. [71] developed a core–shell structure-based approach with a AgNWs@TiO_2_ high-anti-interference flexible capacitive pressure sensor for pulse and respiratory monitoring. They offer instantaneous health feedback to users, enhancing personal health awareness and significantly aiding medical professionals in their diagnostic processes. Furthermore, in the realm of smart healthcare, flexible sensors are instrumental in real-time wound healing assessment, infection prevention, and skin hydration monitoring. The incorporation of flexible sensors into wearable devices and smart healthcare systems is poised to transform the landscape of health management. By delivering a stream of continuous and precise health data, these sensors not only empower individuals to take a proactive stance in their health and well-being but also enable healthcare providers to deliver more efficient and tailored care.

In addition to physiological monitoring, flexible sensors are also making significant strides in chemical sensing applications. Li et al. [76] developed a wearable inorganic oxide chemiresistor based on a flexible Al_2_O_3_-stabilized ZrO_2_ ceramic sponge substrate for NO_2_ sensing. By leveraging the unique properties of flexible materials, these sensors can detect a wide range of chemical parameters, enhancing their utility in various healthcare and environmental contexts.

**Figure 11:**
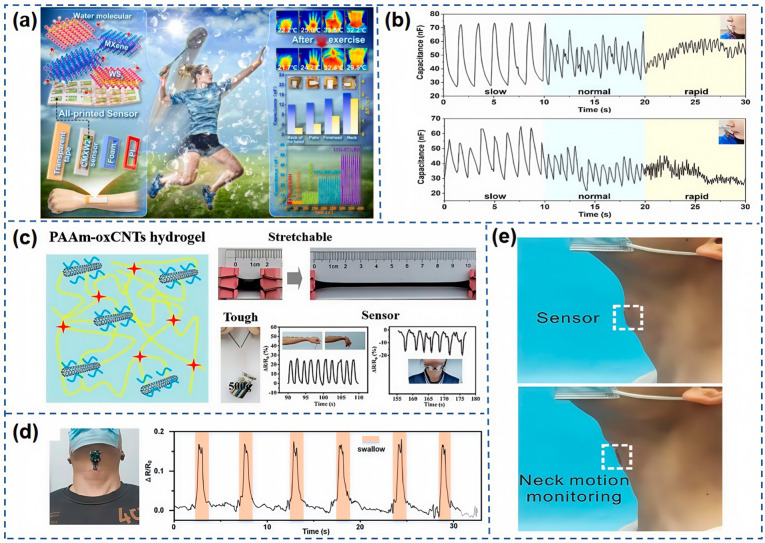
(**a**) Composition of CMXW_2_ humidity sensor, infrared thermal images of temperature changes in the area before and after exercise (such as the back of the hand, palm, forehead, and neck), and comparison of capacitance changes based on sweating in the area [117]. (**b**) The capacitance changes of CMXW2 humidity sensor under slow, normal, and fast oral and nasal breathing [117]. (**c**) Schematic diagram of PAAm oxCNTs hydrogel and images under stretching, used to monitor subtle human movement [118]. (**d**) Data collection of swallowing movements while drinking water [59]. (**e**) CP@GM on the throat of volunteers to monitor the movement of the head, neck, and throat [119].

As shown in Figure 11a, Lu et al. [117] have pioneered the development of a WS_2_-modified MXene composite material for humidity sensing. By blending sodium CMC solution into the MXene/WS_2_ composite, they have fine-tuned the viscosity to formulate a printable hygroscopic ink. Utilizing screen printing technology, they have successfully developed a humidity sensor with rapid response and recovery times of 4.65 s and 1.33 s, respectively, within a RH range of 11–97%. The upper right portion of Figure 11a captures the most pronounced temperature drop following neck movement, as observed by an infrared camera, while the least change is noted on the dorsal surface of the hand. The middle right section of Figure 11a demonstrates the capacitance variations of the CMXW_2_ humidity sensor patch before and after physical activity, corroborating the findings from the infrared thermal imaging. Additionally, this sensor is capable of monitoring respiration in real-time, reflecting the human state, whether at rest or during exercise. Figure 11b presents the response curves of the CMXW_2_ humidity sensor when tracking respiratory activities at the mouth and nose. The trends in capacitance value changes reveal significant waveform differences under varying breathing conditions, from slow to rapid, highlighting the sensor’s potential for skin humidity and real-time respiratory monitoring, thus solidifying its role in smart healthcare applications. In further advancements, Sun et al. [118] have constructed a conductive nanocomposite hydrogel from oxidized MWCNTs and PAM, as detailed in Figure 11c. Zhang et al. [59] have introduced a manufacturing strategy for an AMSS employing 4D printing technology. This sensor, when positioned on the neck, effectively monitors swallowing activities in real-time, as portrayed in Figure 11d.

**Figure 12:**
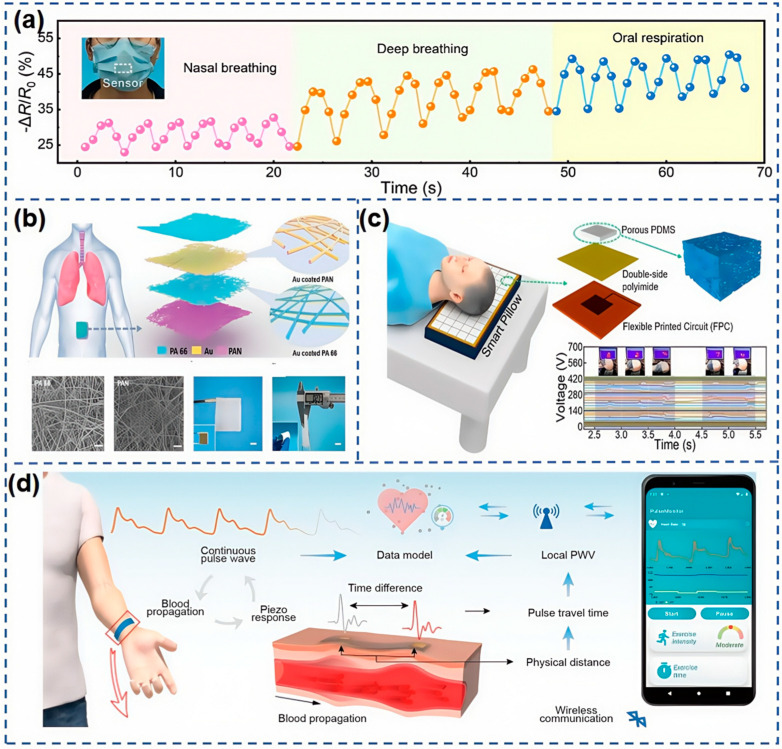
(**a**) Waveforms obtained when volunteers wear the mask correctly to detect nasal, deep, and mouth breathing [119]. (**b**) Structural design and working principle of the TENG-based SANES [120]. (**c**) Schematic diagram of intelligent pillow monitoring head movement [121]. (**d**) Schematic diagram of wearable system monitoring blood pressure [122].

Zhang et al. [119] have developed an innovative cellulose paper-based sensor, CP@GM, which is uniquely responsive to both temperature and pressure. This dual-functional sensor is designed to detect movements in the human head, neck, and throat areas with precision. As shown in Figure 11e, when placed on the throat, the sensor adeptly captures minute skin deformations, translating pressure signals into electrical outputs. It has been tested on six distinct actions: chewing, swallowing, coughing, shaking the head, nodding, and extending the head forward. Subsequent PCA analysis of the sensor’s captured motion signals revealed a clear clustering among the six, indicating the sensor’s ability to effectively discriminate between these movements. This flexibility in detecting neck movements positions the sensor as a promising tool in the smart healthcare sector. Furthermore, the sensor’s capabilities extend to monitoring respiratory conditions during sleep. With growing attention to sleep quality, monitoring sleep respiration is a key metric. The team evaluated four types of breathing behaviors: irregular mask wearing, regular nasal breathing, deep breathing, and mouth breathing. The results showed a pronounced clustering, demonstrating the sensor’s efficacy in distinguishing these behaviors. The waveforms, as shown in Figure 12a, exhibit an overall upward trend in resistance signals due to the sensor’s dual response to pressure and temperature, with resistance changes resulting from their combined effects. Even in cases of irregular mask wearing, the sensor demonstrated high sensitivity in accurately capturing respiratory frequency. Additionally, by monitoring sleep breathing, the sensor can assess sleep quality and promptly identify potential health issues.

Respiratory rate is a critical vital sign for assessing human health, offering valuable insights into an individual’s well-being. As previously discussed, the monitoring of sleep respiration is instrumental in diagnosing sleep disorders. Peng et al. [120] have crafted an electronic skin utilizing TENG, as shown in Figure 12b, which is capable of real-time respiratory frequency monitoring and diagnosing obstructive sleep apnea hypopnea syndrome (OSAHS) by detecting the chest or abdominal expansion and contraction. This electronic skin excels in subtle, real-time detection, showcasing high efficiency. Crucially, it enables real-time monitoring and assessment of the severity of OSAHS, preventing its onset and markedly enhancing sleep quality. Furthermore, Kou et al. [121] have developed an intelligent pillow equipped with a TENG sensing array, which monitors sleep quality by tracking the position and movement of the head during sleep. This intelligent pillow, shown in Figure 12c, is a daring innovation in flexible wearable devices for sleep state monitoring, with vast potential in health and medical surveillance. Blood pressure and pulse are pivotal physiological indicators for gauging cardiovascular health. For chronic hypertension patients, ongoing blood pressure monitoring is an effective strategy for accurately assessing potential health risks, enabling timely and targeted preventative actions to safeguard patient health. Li et al. [122] have introduced a compact system for wearable blood pressure monitoring, shown in Figure 12d. The piezoelectric sensors precisely detect the minute deformations of arteries during blood flow and efficiently transform these into electrical signal outputs. Leveraging sophisticated machine learning models, we can accurately extract key blood pressure characteristics from these voltage signals, including the peaks of systolic and diastolic pressures, achieving Class A measurement precision. This system has successfully addressed a range of challenges in continuous blood pressure monitoring, including system integration, interface performance optimization, and enhancement of measurement accuracy, significantly advancing the application and development of wearable devices in this field.

### 5.3. Motion Monitoring

Motion detection stands out as a key application for flexible sensors, primarily owing to their capacity to adapt to curved surfaces and irregular contours. This flexibility enables them to conform snugly to the human skin, thereby enhancing wearer comfort and bolstering the precision and reliability of monitoring. By embedding flexible sensors within sports gear or affixing them to the wrist, we can accurately capture detailed user movement data. These data are crucial for delivering informed exercise guidance and tailored health management recommendations to users.

**Figure 13:**
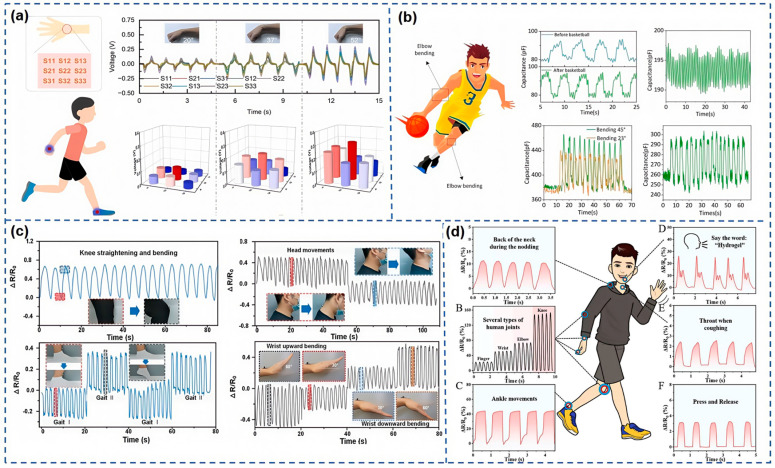
(**a**) Wearable applications for human motion monitoring [123]. (**b**) Application of FCPS device in human basketball motion monitoring [124]. (**c**) Application of AMSS in wearable smart devices for detecting various physiological movements [59]. (**d**) Hydrogel sensors for motion detection in different parts of the human body [3].

Zhen et al. [123] have developed an ultra-thin and flexible piezoelectric sensor that integrates small-pixel, high-sensitivity piezoelectric ceramic thick films into a highly adaptable format. This sensor is designed for monitoring various movements, including joint flexion, human gait, and laryngeal sounds. As shown in Figure 13a, the sensor array is mounted on the wrist, and the magnified image reveals the distribution of nine sensing elements. When the wrist bends, the sensor array deforms, generating a piezoelectric potential. Figure 13a displays the signal output at three different bending angles (20°, 37°, and 52°), with the voltage output increasing as the bending angle becomes larger. The lower right section of Figure 13a shows the voltage output of each individual sensing element, distinguishing different motion states in the corresponding areas. This sensor array demonstrates significant potential for accurately monitoring various movement conditions.

Gao et al. [124] have crafted a flexible capacitive pressure sensor that integrates nylon fabric with PVDF, a combination that yields remarkable sensitivity (33.5 kPa^−1^), an ultra-low detection threshold (0.84 Pa), and a rapid response time (27 ms). This sensor retains its impressive reliability even after enduring over 100,000 cycles of operation. Versatile in its applications, this sensor is adept at monitoring a variety of movements, as demonstrated in Figure 13b, where it is utilized to track the respiration, pulse, and the flexion of arms and thighs of basketball players with precision. Zhang et al. [59] have advanced the field with a manufacturing strategy for an AMSS, leveraging 4D printing technology. This sensor not only monitors swallowing activities in real time but also discerns a range of human movements, as shown in Figure 13c. It is capable of recognizing significant body motions, such as the repetitive bending of knee joints and head movements. Thanks to its high sensitivity, the sensor can also capture intricate human movements, detecting the motion of ankles and wrists with each signal correlating to distinct angles of movement. The sensor’s capabilities are further enhanced by the AMSS’s wireless transmission features, allowing data to be sent wirelessly and displayed on a custom mobile application for the real-time monitoring of various body parts. Wang et al. [3], inspired by the adhesive properties of mussels, have synthesized mesoporous silica @polydopamine-Au (MPS@PDA-Au) nanomaterials and designed a self-healing nanocomposite hydrogel for the detection of human motion. As seen in Figure 13d, when affixed to the skin, this sensor provides real-time monitoring of the body’s motion, from the grand gestures of neck, elbow, and knee movements to the subtle twists of the lips, showcasing superior detection capabilities. Zhang et al. [125] have introduced a non-invasive, sensitive, self-powered triboelectric biosensor (TEBS) that can simultaneously monitor both biochemical and physical aspects of the body in real time. The TEBS demonstrates an extraordinary response capability, detecting glucose, uric acid, and lactic acid at rates of 101%, 64.98%, and 64.80%, respectively. It is also capable of identifying a variety of body movements, including walking, running, squatting, and jumping, making it a powerful tool for comprehensive health monitoring.

### 5.4. Human–Computer Interaction

In the domain of human–computer interaction, flexible sensors have become indispensable, revolutionizing the way we engage with technology. The advent of smart, wearable sensing devices has broken the mold of reliance on keyboards and mice, steering us away from the stiffness and sluggishness of outdated interaction methods. These innovative flexible sensors have paved the way for a more intuitive and natural dialogue between humans and machines, enhancing the fluidity and ease of our digital interactions.

**Figure 14:**
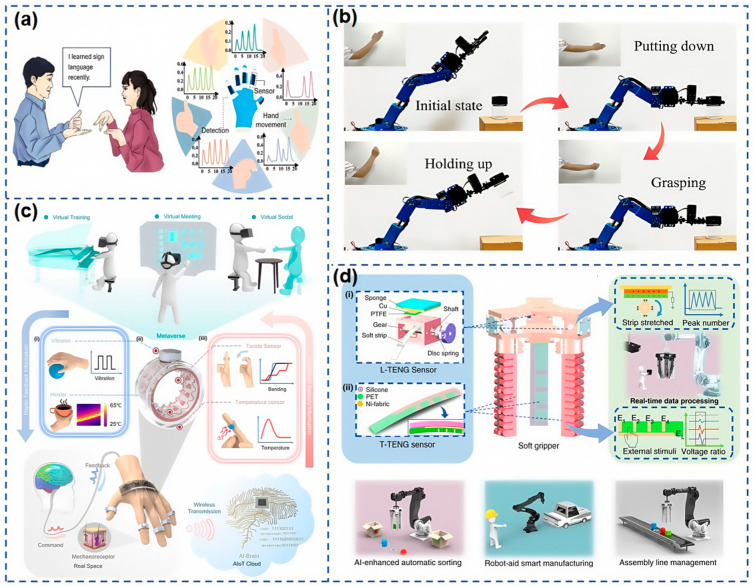
(**a**) Interpretation of sign language images used by deaf and mute individuals [126]. (**b**) Human joint motion manipulation robot arm diagram [127]. (**c**) ATH-Ring based applications, sensing and feedback functions [132]. (**d**) Structural diagram of soft gripper and its digital twin application [133].

In recent years, flexible and stretchable strain sensors have demonstrated immense potential in the field of human–computer interaction, owing to their unique flexibility and high sensitivity. Notably, biomimetic design principles inspired by nature have provided innovative insights for the development of these flexible sensors. Zhang et al. [126] introduced a flexible and stretchable strain sensor inspired by plant scales, known as the LIFSSS. This bioelectronic composite material comprises MWCNTs, graphene, neodymium iron boron, and PDMS. The sensor is designed for use in human–computer interaction to interpret sign language used by the deaf and mute. As shown in Figure 14a, this is achieved through an LSTM network capable of recognizing gestures and generating electrical signals by continuously learning the subtle changes in finger flexion movements. Beyond gesture recognition, the sensor can also be employed for robotic arms, establishing a solid foundation for the precise translation of sign language into robotic actions. Building on this, Wang et al. [127] developed a strain sensor featuring synergistic conductive networks. This sensor consists of a stable carbon nanotube (CNT) dispersion layer and a brittle MXene layer, which are assembled using dip-coating and electrostatic self-assembly techniques, along with a breathable three-dimensional (3D) flexible substrate made from TPU fibers prepared via electrospinning. As shown in Figure 14b, this flexible strain sensor is connected to elbow and joint movements to capture human joint motion data, enabling the manipulation of robotic arms. Consequently, this strain sensor exhibits significant potential for controlling exoskeleton robots.

Wang et al. [128] have crafted a flexible triboelectric sensor array (TSA) with a transverse configuration of sensing units, designed to optimize the human–computer interaction experience. Through intricate patterning and strategic optimization, the TSA achieves high output performance while reducing the number of data channels, streamlining the system for enhanced efficiency. In parallel, Ruan et al. [129] have engineered a flexible 3D force sensor inspired by the biology of octopuses. The CNT/Ecoflex conductive elastomer, which forms the sensor’s body, captures the normal components of 3D spatial forces. Additionally, the transfer of laser-induced graphene sensing film onto Ecoflex (LIG/Ecoflex) emulates the octopus’s tactile tentacles, discerning both the magnitude and direction of the tangential components of 3D spatial forces. This biomimetic sensor stands out for its rapid and efficient response, cyclic stability, and flexible adhesion to the skin, making it ideal for precise handwriting recognition in human–computer interaction scenarios. Shi et al. [130] have developed a piezoelectric organohydrogel that integrates PVDF, acrylonitrile (AN), acrylamide (AAm), p-styrenesulfonate (NaSS), glycerol, and zinc chloride. This material boasts piezoelectric properties, resistance to low temperatures, mechanical robustness, and stable electrical performance, positioning it as a promising candidate for applications in human–computer interaction. Dai et al. [131] have delved into the development of a flexible, self-powered, contactless sensor for human–computer interaction in harsh environments. They have showcased a range of non-contact HMI applications suitable for adverse conditions, including non-contact appliances, mobile trajectory and accidental fall tracking systems, and a real-time machine learning-assisted gesture recognition system with an impressive accuracy rate of 99.21%.

Virtual reality (VR) is a cutting-edge human–computer interaction technology that immerses users in a simulated environment, tricking the senses into believing they are present in an alternate space. This virtual environment is a digitally crafted, computer-generated simulation that mirrors the real world. Users can engage with the virtual environment through a variety of VR devices, such as head-mounted displays and hand sensors, to achieve a truly immersive experience. Sun et al. [132] have introduced augmented tactile haptic rings (ATH-Rings), which, empowered by machine learning analysis, are capable of continuous finger motion detection and sign language translation applications, as shown in Figure 14c. The ATH-Rings excel in their ability to detect finger movements with precision, capturing the subtlest of gestures and analyzing them using sophisticated machine learning algorithms. This enables a range of efficient human–machine interface (HMI) applications, including the translation of sign language. The incorporation of haptic feedback into the ATH-Rings significantly enhances the immersive experience of VR applications, providing users with a more realistic and vivid interaction within the virtual world, making them feel as if they are genuinely immersed in the experience. Building on this, Jin et al. [133] have crafted even more intricate digital spaces within VR environments. They have successfully integrated TENG-based sensors into soft grippers and effectively synthesized information from multiple sensors using machine learning techniques, leading to the efficient and accurate recognition of target objects, as shown in Figure 14d. To offer a more intuitive demonstration of this process, they have also developed a digital twin virtual environment. This maps the sensor perception and recognition capabilities into the digital realm, enabling the twin model to carry out intelligent sorting operations. This innovation paves the way for new applications and solutions in the field of intelligent manufacturing for flexible robots, particularly in scenarios that require the integration of multi-dimensional information.

### 5.5. Summary

This chapter delves into the myriad applications of flexible sensors across a spectrum of domains, including electronic skin, healthcare monitoring, motion tracking, and human–computer interaction, highlighting the extensive reach and significant potential of this cutting-edge technology. The development of flexible sensors tailored to diverse scenarios through various sensing mechanisms and material choices represents a burgeoning trend. Electronic skin, a novel class of flexible wearable sensors, emulates the sensory functions of human skin with its exceptional flexibility, transparency, and biocompatibility. Thanks to innovative designs and material selections, electronic skin can conform closely to the human body or robotic surfaces, providing multifaceted perception of the external environment, and holds vast promise for applications in medical monitoring and human–computer interaction.

Flexible sensors are increasingly being integrated into the healthcare sector, offering real-time monitoring of vital signs such as heart rate, blood pressure, and respiratory rate, which furnish physicians with precise diagnostic insights. Moreover, these sensors extend their utility to wound monitoring and skin hydration assessment, thereby providing holistic support for medical care. In the realm of motion tracking, flexible sensors play an indispensable role. Their ability to conform snugly to the skin and enhance wearer comfort allows for the precise capture of user movement data, which is instrumental in scientifically guiding exercise regimens, enhancing workout efficacy, and preventing sports-related injuries. Within the sphere of human–computer interaction, flexible sensors have transformed the interaction between humans and machines into a more intuitive and natural experience. Innovative designs of flexible sensors enable interaction through gestures, voice, and other modalities, significantly improving the convenience and efficiency of engagement. Furthermore, the integration of flexible sensors in VR and augmented reality (AR) applications has ushered in a new era of immersive experiences for users.

The ongoing evolution of flexible sensor technology is bringing about transformative changes in multiple sectors. With the continuous advancements in material science, micro-nanofabrication, and information technology, the capabilities of flexible sensors are poised to enhance, and their applications are set to broaden. Notably, leading research groups around the world are driving these advancements.

The flexible strain sensor based on graphene/carbon nanotube composites developed by Stanford University’s Bao Zhenan team has high sensitivity, fast response time, and good durability [134]. Ali Javey’s team at the University of California, Berkeley, USA, has achieved a breakthrough in the performance of flexible sensors in terms of high sensitivity, fast response and low detection limit by improving the fabrication process and device structure of nanowires [135]. The triboelectric nanogenerator-driven flexible sensor developed by Wang Zhonglin’s team at the Institute of Nanoenergy and Systems of the Chinese Academy of Sciences can convert mechanical energy into electrical energy and provide self-drive power for the sensor, realizing self-drive sensing without external power [136].

## 6. Challenges and Outlook

**Figure 15:**
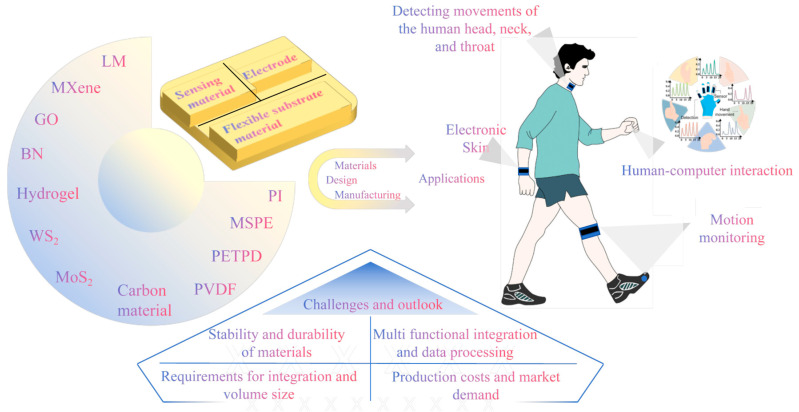
Flexible sensor materials and their applications and challenges in wearable devices.

This article takes flexible sensors as its focal point, beginning with their evolutionary history and delving into the “sensing mechanisms–materials–applications” paradigm across different fields of application, offering an exhaustive review of the recent developments in the field. Flexible sensors, known for their pliability and stretchability, can be freely bent, folded, and twisted, conforming to various complex surfaces. Crafted from lightweight materials, they are portable and easy to install. Moreover, flexible sensors possess advantages such as high sensitivity, multimodal monitoring, and swift response times. These distinctive attributes are amplifying the benefits of flexible sensors, providing substantial reference value for the advancement of a wide array of industries. Naturally, the effectiveness of flexible sensors in applications is often contingent upon their sensing mechanisms, material selections, and the specific application context. In recent years, a diverse array of flexible materials has come to the forefront, including metallic materials, CNTs, graphene, carbon fibers, MXene 2D materials, hydrogels, and their composites. It is noteworthy that the fabrication of flexible sensors primarily hinges on the development of composite materials, capitalizing on the strengths of various materials to optimize the performance of flexible sensors. This article concentrates on and synthesizes the latest research advancements in electronic skin, health and medical monitoring, motion tracking, and human–computer interaction applications, as shown in Figure 15. While flexible sensors have demonstrated immense potential and made significant strides in the realm of smart wearables, they also confront certain challenges amidst the trend towards large-scale industrialization:

(1) For flexible sensors that are in constant contact with the human skin, the stability and durability of the materials are of paramount importance. Yet, some flexible materials may face issues of performance degradation or damage over extended periods of use, with these issues being particularly exacerbated in harsh environmental conditions. Consequently, the development of innovative, eco-friendly flexible materials that can improve the sensors’ stability and longevity is a critical area of focus for future research endeavors.

(2) With the ongoing miniaturization of smart wearable devices, there is a growing demand for flexible sensors that are not only compact but also maintain their sensitivity and precision. The challenge lies in downsizing these sensors without compromising their performance, ensuring they continue to offer high accuracy and sensitivity even in complex environments. This balance is a critical technical hurdle that must be overcome.

(3) With deepening research, flexible sensors are poised to embrace an array of advanced features, such as self-healing, self-powering, and biocompatibility. A critical issue for the future is the integration of numerous electronic components and sensors onto diminutive soft substrates, which demands large-scale, cost-effective integration and manufacturing techniques. Concurrently, the proliferation of multifunctional sensors will yield substantial electrical signal data, and the collection and processing of this information to ensure the sensors’ reliable operation present a formidable challenge.

(4) Prior to the design and fabrication of flexible sensors, their production costs must be taken into account as the current high costs associated with manufacturing such sensors have, to a certain degree, hindered their widespread adoption. A pressing priority is the need to reduce these costs while maintaining performance standards to facilitate large-scale production. Additionally, market demand poses a significant challenge, with varying requirements for flexible sensors across different sectors. It will be essential to gain a thorough understanding of market demands in the future and to develop sensors that cater to diverse application scenarios and even pursue the development of “multi-functional devices”.

## Figures and Tables

**Table 1:** Comparison of flexible sensors with three sensing mechanisms.

Classify	Materials	Detection Range (KPa)	Sensitivity (Kpa^−1^)	Cyclic Stability (times)	Refs.
Flexible piezoresistive sensor	PDMS, MWCNT, PVDF	1.58–600	0.06	10,000	[12]
	MXene, olyacrylate tape	0–16	148.26	13,000	[95]
	PANI, SiO_2_, silk fibroin	10–380	0.071	2000	[103]
	macro-bamboo fiber, Go	0–300	892.9	7000	[104]
	CNT/LM, PDMS/Ecoflex, copper wires	5–30	-	1000	[105]
Flexible capacitive sensor	Aprotic/protic ionic liquids, PAM, LiCl	70–900	2.75	50,000	[106]
	polyvinyl alcohol (PVA), phosphoric, AL, PDMS	1–1000	0.54	-	[69]
	PVA/sodium alginate,	0–300	0.54	5000	[70]
	graphene nanowalls, PDMS, ZnO	0–22	22.3	2000	[107]
Flexible triboelectric sensor	Activated carbon/PU, PET,	0–10	0.94	1300	[108]
	expanded polytetrafluoroethylene membranes, paper substrate, nylon fabric, Cu electrode	0–400	-	10,000	[109]
	texlile, CNTs, fluorinated ethylene propylene, AI, PDMS	-	0.21	10,000	[110]

## Data Availability

All data generated or analyzed during this study are included in this study.

## Nomenclature

PI	Polyimide	PET	Polyester	PVDF	Polyvinylidene fluoride
PU	Polyurethane	ZnO	Zinc oxide	PDMS	Polydimethylsiloxane
TSA	Triboelectric sensor array	TiO_2_	Titanium dioxide	PEDOT	poly(3,4-ethylenedioxythiophene)
LM	Liquid metal	RH	Relative humidity	N-MXene	MXene Ti_3_C_2_T_x_
CNTs	Carbon nanotubes	BA	Butyl acrylate	PEEK	Polyether ether ketone
AMSS	Adaptive multifunctional strain sensor	2D	Two-dimensional	ATH-Rings	Augmented tactile haptic rings
AR	Augmented reality	TPU	Thermoplastic polyurethane	MWCNTs	Multi-walled carbon nanotubes
HMI	Human–machine interface	SMC	Symmetric miura-ori capacitive	AgNWs	Silver nanowires
VR	Virtual reality	TENG	Triboelectric nanogenerators	GPLA	Graphene polylactic acid
MoS_2_	Molybdenum disulfide	PEI	Polyethyleneimine	PLA	Polylactic acid
BN	Boron nitride	CNF	Cellulose nanofiber	OSAHS	Obstructive sleep apnea hypopnea syndrome
CT–LM	Chromium telluride-coated liquid metal	FECTs	Organic electrochemical transistors	CFRSMCs	Carbon fiber-reinforced shape memory composites
PVA	Polyvinyl alcohol	ASZ	Al_2_O_3_-stabilized ZrO_2_	CMC	Carboxymethyl cellulose
PANI	Polymerized polyaniline				
